# Preventing smoking initiation among vulnerable adolescents: a process evaluation of the KickAsh!-intervention in Flemish youth social work settings

**DOI:** 10.1186/s13690-026-01972-6

**Published:** 2026-06-05

**Authors:** Babette Demeester, Sara Willems, Kenji Leta, Peter A. J. Stevens, Maïté Verloigne, Emelien Lauwerier

**Affiliations:** 1 Department of Public Health and Primary Care, Ghent University, Sint- Pietersnieuwstraat 33, Gent, 9000 Belgium; 2 Department of Social Sciences, Ghent University, Sint-Pietersnieuwstraat 33, Gent, 9000 Belgium; 3 Department of Psychology, Open University, Valkenburgerweg 177, Heerlen, 6419 AT the Netherlands

**Keywords:** Health promotion, Co-creation, Process evaluation, Smoking prevention, Adolescents, Low socioeconomic status, Youth workers

## Abstract

**Background:**

Smoking disparities remain a global concern, with adolescents experiencing societal vulnerability at increased risk of cigarette smoking initiation. Persistent inequities suggest that prevention interventions often fail to engage these adolescents, partly because they insufficiently reflect their everyday environments and social contexts. Although schools are commonly used settings, their impact is often limited for this group, underscoring the need for more context-sensitive approaches and to explore complementary settings. To address this, the KickAsh!-intervention was co-created with adolescents and youth workers within youth social work organisations. In an accompanying paper, the effectiveness study of KickAsh! showed no significant effects, highlighting the need to examine how the intervention was implemented in practice. This study therefore presents a process evaluation, aiming to understand the mechanisms underlying its (lack of) effects and how implementation interacts with context in real-world settings.

**Methods:**

KickAsh! was implemented across eight youth social work organisations. Qualitative data were collected through seven focus groups with adolescents (*n* = 52) and eight interviews with youth workers (*n* = 8). Framework analysis enabled systematic organisation of data, cross-case comparison, and identification of deeper themes.

**Results:**

Seven themes emerged: (1) perceptions of the intervention and its impact, (2) preferences and the importance of adaptability, (3) influence of youth workers’ smoking status, (4) influence of their skills and motivation, (5) organisational structure and context, (6) addressing smoking within youth social work, and (7) participation and co-creation.

**Conclusion:**

Implementation of the KickAsh!-intervention varied substantially across youth social work organisations due to interacting individual, organisational, and intervention-related factors, highlighting its strong context dependency. This variability may explain the limited observed effects, although the intervention appeared to foster important unmeasured outcomes such as awareness, open dialogue, and organisational capacity. The development and refinement of a theory of change further underscored that inconsistent implementation and limited fidelity were key drivers of these findings, alongside broader contextual influences. Youth workers played a pivotal role, as their characteristics, skills, and capacity largely determined success. Embedding flexibility to adapt the intervention to each organisation’s specific context and needs appears crucial for feasibility, impact, and sustainability.

**Trail registration:**

This study is part of a larger project that includes the development, implementation and evaluation of the KickAsh!*-*intervention. The study protocol of this project is registered on ClinicalTrials.gov (NCT05920772).

**Supplementary Information:**

The online version contains supplementary material available at 10.1186/s13690-026-01972-6.

**Table:** 

Text box 1. Contributions to literature
• Youth social work organisations all have their unique context, influencing how a smoking prevention intervention will be implemented and perceived.
• Personal background and characteristics of youth workers will influence implementation.
• Youth workers need sufficient guidance on how to adapt a smoking prevention intervention to their unique context without losing core elements.
• Adolescents and youth workers should be involved when planning implementation and evaluation of a smoking prevention intervention.
• KickAsh! supported youth workers in making smoking more discussable. However, smoking is still sometimes perceived as sensitive, highlighting the need to further strengthen youth workers in addressing this topic within their organisations.

## Background

Cigarette smoking among adolescents remains a critical global health issue. Approximately 5% of the 13-to 15-year-olds smoke cigarettes, representing an estimated 20 million young people worldwide [1]. In Flanders (Belgium), nearly one in five adolescents aged 12 to 18 report having ever smoked a cigarette, and 14.6% report smoking in the past year [2]. These are alarming numbers, as adolescents are more vulnerable to nicotine addiction because of ongoing brain development, increasing their risk of dependency and developing chronic diseases in the long term, such as cancer and cardiovascular conditions [3, 4]. Adolescents experiencing societal vulnerability (e.g. poverty, minority status, migration background) are at an even greater risk, partly due to increased exposure to smoking in their social environment (including family members), living in neighbourhoods with a higher density of tobacco retailers, and higher stress levels [5–8]. For these adolescents, the health consequences of smoking can be more severe, with multiple risk factors exacerbating adverse outcomes over time [9]. Persistent smoking-related health inequities indicate that current prevention interventions (i.e. targeted interventions to prevent people from starting to smoke) fail to reach or engage adolescents experiencing societal vulnerability [10]. These programmes may be insufficiently attuned to adolescents’ everyday environments and social contexts, limiting their relevance and impact. Accordingly, public health research increasingly emphasises the importance of contextual sensitivity, as the setting in which an intervention is implemented not only shapes its delivery but also influences its effectiveness [11, 12]. Although schools are commonly used as intervention settings due to their accessibility, the impact of school-based programmes varies and is often limited for adolescents with weaker connections to school or whose social realities and daily environments are not adequately reflected in these interventions [13–16]. As argued by Vettenburg, adolescents experiencing societal vulnerability are more likely to encounter the sanctioning dimensions of school and other societal institutions and some of them experience less constructive relationships with institutional actors (e.g. teachers), which may undermine engagement in interventions implemented in these contexts [15]. It is therefore important to identify complementary settings and contextual conditions that can meaningfully engage adolescents experiencing societal vulnerability in smoking prevention, rather than framing prevention efforts as situated exclusively within or outside the school context. In this regard, youth social work (YSW) organisations present a promising and underexplored setting, given their explicit focus on adolescents experiencing societal vulnerability and their capacity to offer a supportive, trust-based context which may enhance engagement in smoking prevention efforts. YSW organisations provide leisure-time activities that aim to empower young people by expanding their living environments, fostering group cohesion, reducing barriers to participation, and strengthening social skills [15, 17, 18]. Moreover, YSW places a strong emphasis on adolescents’ physical and psychosocial well-being, rendering it a highly relevant context for health promotion initiatives [17, 19, 20]. In addition, youth workers can play a pivotal role in delivering effective smoking prevention messages and fostering empowerment, as they often build strong relationships with adolescents and may even serve as role models [21]. Importantly for this study, smoking is prohibited in most indoor and child-oriented public spaces in Belgium; however, no uniform regulations apply to YSW, leaving organisations to define their own smoking policies. This results in variation in norms and practices across the sector.

Therefore, to address cigarette smoking initiation among adolescents experiencing societal vulnerability, the KickAsh!-intervention was developed: a smoking prevention programme designed for implementation within YSW organisations. As the mean age to start smoking in Belgium is 15.2 years old [2], the intervention specifically targets adolescents aged 10 to 16 years old. KickAsh! seeks to influence smoking initiation by targeting several determinants. These determinants were addressed using theory-based methods [22, 23] which were translated into different intervention components. Most components were directed at the individual level, including self-efficacy and coping planning, attitude, risk perception, perceived social pressure and subjective norms. It consists of different components, of which most are directed at adolescents themselves (i.e. mood boards, smoke-free games, a smoke-free camp, and the KickSomeAsh!-challenge). Other components aim to influence adolescents indirectly by shaping their environment (i.e. outcomes on the environmental level), including adjustments to the smoking policy within YSW organisations and providing youth workers with practical tips and strategies to address smoking in their settings. An overview of the intervention components is presented in Supplementary Material 1. To enhance both its effectiveness and feasibility, the intervention was created using a co-creative approach, allowing it to be tailored to the specific needs of adolescents by actively involving them throughout the development process [24, 25]. Youth workers were also involved in the co-creation process, given their expertise in engaging with adolescents in these settings [16]. Moreover, as they serve as the primary implementers of the intervention, it was essential to consider their specific needs and perspectives regarding implementation. A detailed explanation of the co-creation process and how it resulted in KickAsh! is available elsewhere [26].

A crucial step in intervention research is evaluation. It is now well-accepted that it is not only important to determine whether an intervention works, but also how it works, meaning an understanding of the intervention’s underlying mechanisms in relation to the context where it is implemented [11, 27]. The findings of our own effectiveness study clearly illustrate this need. As outlined in the study’s protocol, the effectiveness of the KickAsh!-intervention was assessed through a non-randomised cluster-controlled trial [28]. The trial only revealed significant effects for coping planning (i.e. preparing strategies to resist smoking), and not for smoking initiation or other related determinants [29]. These findings raise important questions about the underlying reasons for the limited effectiveness. More broadly, they illustrate that relying solely on quantitative outcome measures may be insufficient to fully evaluate complex interventions (i.e. interventions comprising multiple interacting components, targeting diverse organisational levels, and often posing implementation challenges [12]) such as KickAsh!. Therefore, a thorough process evaluation is needed to provide deeper insight into the mechanisms driving intervention outcomes. Grounded in established frameworks such as the Medical Research Council (MRC) guidance for process evaluation of complex interventions [11, 12], this evaluation approach helps to clarify how and why interventions succeed or fail, why unexpected outcomes occur, and how implementation processes interact with contextual factors. Ultimately, this contributes to the refinement and development of more effective interventions [11, 12, 27]. The findings from such an evaluation can provide relevant insights for future research, both within the specific context of YSW and in the broader field of smoking prevention.

This study aims to conduct a process evaluation of the KickAsh!-intervention, a co-created cigarette smoking prevention intervention targeting adolescents experiencing societal vulnerability. More specifically, we seek to understand the mechanisms and processes behind the intervention’s (lack of) effect and to explore the interactions between the intervention and the context of its implementation, i.e. YSW organisations. By doing so, the study addresses a gap in the literature regarding the feasibility of implementing smoking prevention interventions within the under-researched YSW context. As such, we aim to generate insights into how complex interventions are adapted and delivered in real-world community settings, which will not only strengthen the KickAsh!-intervention but also inform the development of similar initiatives. Accordingly, the study analyses the perspectives of both adolescents, as the target population, and youth workers, as the key implementers of the intervention.

## Methods

### Study design

This study is part of a larger project that includes the development, implementation and evaluation of the KickAsh!*-*intervention. The study protocol of this project was previously published [28] and registered on ClinicalTrials.gov (NCT05920772). While the effectiveness of the intervention is addressed in a separate publication [30], the present study focuses on the process evaluation. This evaluation primarily draws on qualitative data, collected through focus groups with adolescents and interviews with youth workers, all conducted after the implementation phase of the intervention.

### Implementation procedure of KickAsh!

To ensure accessibility and ease of use for implementers, i.e. youth workers, the KickAsh!-intervention was compiled into a digital toolkit in the form of a website. This website was exclusively intended for youth workers and provided access to all necessary materials, both digital and physical, along with detailed implementation instructions. Given the diversity in objectives and used methods across YSW organisations (e.g. organisations offering sports or focusing on recreational activities), youth workers were allowed to adjust the intervention components (i.e. mood boards, tips & tricks for youth workers, policy guidelines, games, KicksomAsh-challenge, and smoke-free camp) to their specific context, provided the core content remained unchanged. For example, one of the games included statement cards; youth workers could modify the game’s format as long as these cards were used, since they represent the core element that directly targets the intended determinants. An overview of the core elements for each intervention component is provided in Supplementary Material 1. This flexibility was intended to enhance the intervention’s feasibility and relevance within different settings [31]. During the implementation phase of the intervention, youth workers were instructed to implement all components of the intervention, except for the smoke-free camp. This component, which integrates several other elements, was considered optional as it might not align with the timing or capacity of every organisation.

Before implementation, each youth worker attended an in-person meeting where the intervention was explained and demonstrated. The website was shown, and all physical materials (i.e. mood boards, statement cards, ID cards) were distributed. Youth workers also received an on-paper folder with the website link, an overview of the intervention sequence, detailed game instructions, and supplementary materials (e.g. rebus, cigarette-butt page). We encouraged them to contact us with any questions during implementation. They also received a digital logbook to document their implementation activities, including who implemented each component, the number of adolescents present, barriers and facilitators, deviations from the initial implementation plan, and general reflections. Midway through the implementation period, we held an online follow-up meeting with each youth worker to discuss progress and address emerging issues.

### Recruitment & sample

Approximately 70 YSW organisations in Flanders, Belgium, were invited to participate in the evaluation of the KickAsh!-intervention, of which the process evaluation is a part. Detailed information regarding the recruitment strategy and allocation process of these organisations can be found in the project’s study protocol [28] and effectiveness study [30]. Ultimately, 23 YSW organisations agreed to participate, with 11 assigned to implement the intervention (i.e. the intervention group) and the remaining 12 serving as a control group for the effectiveness study. Participants for the process evaluation were recruited exclusively from the intervention group. During the implementation period, three organisations from the intervention group withdrew, resulting in eight organisations participating in the present study. Of these, six primarily focus on organising recreational activities (RA) after school, while two are more oriented toward sports-based activities (SA). Two of the participating organisations were also involved in the co-creative development of the KickAsh!-intervention.

To collect the qualitative data, a focus group discussion was conducted with adolescents in each YSW organisation. Adolescents were recruited on site: researchers visited each organisation and invited the adolescents who were present on that day to participate in the focus group. Consequently, adolescents were not mixed across organisations but only interacted with peers from their own organisation. To maximise the likelihood that adolescents would be present on the day of the focus group, researchers deliberately scheduled visits on busy days (e.g. Wednesday afternoons). Youth workers were also asked to promote the focus group in advance and to remind adolescents, particularly those who had participated in the intervention, to attend on the day of the interview. Inclusion criteria for adolescents included being between 10 and 16 years old, having sufficient understanding of the Dutch language and having participated in the activities offered by the YSW organisation during the implementation period. In total, 52 adolescents from seven different YSW organisations participated in this study. It was not possible to conduct interviews with adolescents from the eighth organisation, as youth workers reported significant resistance among the adolescents and subsequently decided to discontinue the implementation of the intervention and adolescents’ participation in the study. Sociodemographic information of the adolescents who participated in the focus groups can be found in Table 1.

**Table 1:** Socio-demographical characteristics of adolescents participating in process evaluation of KickAsh!

Characteristics		Adolescents (*n* = 52)
*Gender n (%)*	Girls Boys	22 (57.7) 30 (42.3)
*Age*	Mean (SD) Min - Max	13.3 (± 2.2) 7.3–17.7
*Type of YSW organisation n (%)*	Recreational activities (RA) Sport activities (SA)	37 (71.1) 15 (28.9)
*YSW organisation n (%)*	1 2 3 4 5 6 7 8	6 (11.5) 4 (7.7) 4 (7.7) / 10 (19.2) 8 (15.4) 13 (25.0) 7 (13.5)
*Attendance in YSW organisation n (%)*	Every day A few times a week Once a week A few times a month One or two times a month Never/it’s the first time	11 (21.2) 24 (46.2) 10 (19.2) 3 (5.8) 3 (5.8) 1 (1.9)
*Education type n (%)*	Primary education General track Technical track Vocational track Artistical track Not reported/don’t know	14 (26.9) 13 (25.0) 5 (9.6) 18 (34.6) 1 (1.9) 1 (1.9)
*Birth country n (%)*	Belgium The Netherlands Marrocco Turkey Other	39 (75.0) 2 (3.8) 1 (1.9) 2 (3.8) 8 (15.4)
*Birth country father n (%)*	Belgium Marrocco Turkey Other Not reported/don’t know	22 (42.3) 4 (7.7) 6 (11.5) 19 (36.5) 1 (1.9)
*Birth country mother n (%)*	Belgium Marrocco Turkey Other	20 (38.5) 5 (9.6) 7 (13.5) 20 (38.5)
*Education level father n (%)*	No education Primary education Secondary education Higher education Not reported/don’t know	2 (3.8) 1 (1.9) 7 (13.5) 10 (19.2) 32 (61.5)
*Education level mother n (%)*	No education Primary education Secondary education Higher education Not reported/don’t know	/ 2 (3.8) 7 (13.5) 12 (23.1) 31 (59.6)
*Employment father n (%)*	Employed Not currently employed (e.g. sick, retired, housekeeper, caretaker…) Not reported/don’t know	43 (82.7) 2 (3.8) 7 (13.4)
*Employment mother n (%)*	Employed Not currently employed (e.g. sick, retired, housekeeper, caretaker…) Not reported/don’t know	38 (73.1) 11 (21.2) 3 (5.8)

In each organisation assigned to the intervention group, one youth worker was responsible for implementing the intervention. These youth workers were also recruited for participation in an individual interview. In total, eight youth workers were interviewed, of whom five identified as male (62.5%). The mean age (SD; range) of the youth workers was 27 years (± 2.8; 25–33). Three youth workers had never smoked, two were current smokers, and three had smoked in the past but were current non-smokers.

### Focus groups & interviews

To conduct the process evaluation, seven focus groups with adolescents were conducted, with group sizes ranging from four to thirteen participants. Given previous experiences indicating that traditional focus group formats (i.e. where participants sit around a table and respond to questions) are often less effective with adolescents experiencing societal vulnerability [32], more engaging and participatory methods were employed. Therefore, the interview guide used for the focus groups with adolescents (see Supplementary Material 2) differed from what was originally proposed in the study protocol [28]. Each focus group consisted of three parts: (1) All intervention components were printed on large sheets of paper and adolescents were asked to place emoji stickers representing different emotions (e.g. a laughing emoji to indicate enjoyment) on the components. This was followed by a group discussion reflecting on the choices made (2). Adolescents responded to a series of statements by physically indicating agreement or disagreement (e.g. standing up, sitting down, raising a hand). Each statement was followed by a group discussion, with additional probing questions based on participants’ responses. Statements included: “I think it is good when youth workers smoke in [YSW organisation].”, “I already had a conversation with one of the youth workers.”, “If someone offers me a cigarette, I dare to say ‘no’.”, and “I think it is fun to learn about smoking in [YSW organisation].” (3). To conclude, adolescents were asked what they would do if they had 10 million euros to create their own smoking-related project or improve the current one. As a small incentive, participants received a chocolate cookie for their involvement in the focus group.

For the interviews with youth workers, an interview guide (see Supplementary Material 3) was developed covering the following themes: (1) relevant contextual factors 92), the general implementation process (3), delivery and fidelity of the intervention (4), feasibility of the intervention (5), perceptions and experiences concerning the intervention (6), recommendations for future organisation of the intervention (7), impact of the intervention. These themes differ slightly from those originally outlined in the study protocol [28], as a programme theory was developed and refined throughout the course of the study, and the interview guide was adapted to align with this evolving programme theory. Such a theory enhances the reliability and relevance of predicting and evaluating the intervention success [33]. The full programme theory is provided as a supplementary file (see Supplementary Material 4).

Focus groups and interviews were conducted primarily by the lead researcher of the study (BD), with a second researcher (KL) present as an observer. All interviews took place at the respective YSW organisation. Both focus groups and interviews were audio-recorded and transcribed verbatim. The researchers were both young adults, making it easier to empathise with the participants, especially with youth workers. Additionally, both were actively involved in the co-creative development of the KickAsh!-intervention, providing them with in-depth knowledge and contextual understanding of its components and objectives. One of the researchers identified as female, while the other researcher identified as male. Both researchers were trained in qualitative research methods and had experience with conducting interviews and qualitative research [16, 32]. Adolescent participation during the focus groups varied across YSW organisations. In some cases, adolescents were highly engaged, actively contributing to the discussion and eager to share their experiences. In other instances, they appeared distracted, disengaged, or interacted playfully with one another, which occasionally hindered the flow of the conversation. Interviews with youth workers always went smoothly, with participants demonstrating a high level of engagement.

### Analysis

The study protocol initially specified the use of qualitative content analysis (QCA) for analysing the process evaluation data. However, we opted for framework analysis [34–36] instead, as this approach was better aligned with the study objectives. Positioned between coding reliability approaches and the reflexive approach [37], framework analysis enables the systematic organisation of data within a framework matrix. This matrix consists of rows, representing cases (i.e. the participating YSW organisations), columns (i.e. codes) and cells containing summarised data. This structure facilitates both within-case analysis (i.e. focusing on individual YSW organisations as units of analysis) and cross-case analysis (i.e. seeking patterns across YSW organisations), thereby supporting the development of deeper themes. In addition to enabling systematic comparison across cases, framework analysis was considered particularly suitable because QCA is primarily descriptive. As the study progressed, it became clear that a more interpretative and reflexive analytical approach was needed to capture the complexity of both the YSW context and the data. Framework analysis provided the necessary balance between structure and interpretative depth, allowing for the identification of underlying mechanisms and patterns beyond surface-level description.

First, familiarisation with the data took place by listening to the audio recordings of the interviews and reading the transcripts, taking notes of meaningful results. Then, a deductive approach was used to index the data. Codes were pre-defined based on the framework developed by Longworth et al. [38], which identifies key components of process evaluation, specifically for co-created interventions. This framework consists of six overarching dimensions (i.e. delivery, participation, experience, context, maintenance and impact), each encompassing different subcomponents of process evaluation. All relevant components were included as codes for the analysis. Nonetheless, researchers retained the flexibility to introduce additional codes inductively when relevant insights emerged from the data.

To enhance the credibility and trustworthiness of the analysis, two researchers (BD and KL) independently coded two interviews with youth workers and one focus group with adolescents. The outcomes of this initial coding exercise and possible discrepancies between the two coders were discussed with the research team (BD, KL, EL, MV, SW and PS), and adjustments to the codes or indexing strategy were made as necessary. Subsequently, all transcripts were indexed by the lead researcher (BD). The final coding scheme, referred to as ‘index’ in framework analysis, is available as a supplementary file (see Supplementary Material 5). Next, a framework matrix was generated, charting the coded data into the matrix. To facilitate a more focused analysis, a separate matrix was created for each dimension of process evaluation (cf. framework of Longworth et al. [38]). These matrices summarised the data per case (i.e. per YSW organisation), providing a structured overview of how youth workers and adolescents perceived the subcomponents within each process evaluation dimension. To further enhance the credibility of the analysis, different members of the research team (KL, EL, MV, SW, and PS) each reviewed a subset of cases, together covering the entire dataset. This process resulted in the formulation of three main themes (context, implementation, and experiences/impact), which subsequently served as the analytical lens for the data. Through iterative discussions within the research team, these themes were further explored, allowing for deeper insights and a more nuanced understanding, ultimately leading to the final synthesis of the data.

Several strategies were used to strengthen qualitative rigour. The lead researcher (BD) documented all analytic decisions to ensure transparency and traceability. During analysis, we applied validity-enhancing techniques such as the constant comparative method and deviant case analysis. Data triangulation was supported by multiple data sources, including field notes, process evaluation questions added to the effectiveness questionnaire [30], and youth workers’ logbooks. These additional sources were used to enrich contextual understanding during analysis but were not included as empirical results in this study. Furthermore, the credibility and trustworthiness of the findings were strengthened through double-coding a subset of data and involving the full research team at various stages of analysis, as described above. The team’s diverse backgrounds helped reduce bias and enrich interpretation. Finally, reflexivity was embedded throughout the study through reflexive writing and regular team meetings to discuss progress, challenges, and emerging insights.

Nvivo 12 was used to index the data. Microsoft Excel was used to develop the framework matrices (available upon request). The COREQ (Consolidated criteria for Reporting Qualitative research) checklist was consulted and added in Supplementary Material 6 [39].

## Results

The implementation process and usage of the intervention varied significantly across the different YSW organisations. These differences were largely due to the unique contexts of each organisation and the complex interplay of multiple influencing factors that shaped the implementation. These specific factors and the unique context for each YSW organisation are presented in Table 2. To further structure the findings of this study, the results were divided into seven main themes: (1) general perceptions of the intervention and its perceived impact (2), preferences regarding the intervention and the importance of adaptability (3), the influence of youth worker’ smoking status (4), the influence of youth workers’ skills and motivation (5), the influence of the organisation and structure of YSW organisations (6), perspectives on addressing the topic of smoking within YSW, and (7) the influence of participation and co-creation.

**Table 2:** Contextual factors and implementation process per youth social work organisation (process evaluation of KickAsh!)

	1	2	3	4	5	6	7	8
Context								
RA or SA organisation	RA	RA	RA	RA	RA	RA	SA (football)	SA (multi sport)
Current smoking policy	Smoking is prohibited in and around the organisation.	Smoking is prohibited; if YW sees someone smoking, he will talk about the health consequences and ask them to leave.	Smoking is prohibited, but YW agreed that adolescents can vape/smoke outside and on the terrace if he does not see it.	Smoking is prohibited, but adolescents can smoke ‘around the corner’.	Smoking is prohibited; if YW sees someone smoke, they will have a conversation about why it is prohibited.	No explicit rules about smoking.	Smoking is prohibited.	Smoking is prohibited.
Part of co-creation?	Yes	No	No	No	Yes (only YW)	No	Yes	No
Smoking status YW	YW smoked in the past, but stopped.	YW smokes; he believed it to be hypocritical to proclaim not to smoke if he smokes himself.	YW does not smoke, which made it more difficult to talk about this subject with adolescents.	YW smokes; some adolescents know this, but he does not perceive this as a problem.	YW smoked in the past, but stopped.	YW smoked in the past, but stopped. Adolescents often see other youth workers smoke at the organisation	YW does not smoke.	YW does not smoke.
Motivation YW / adolescents	YW was very motivated.	YW was very motivated.	YW experienced resistance from adolescents and could not motivate them to participate in the intervention.	YW was not able to motivate adolescents to participate in the intervention.	YW was motivated to implement, but implementation sometimes felt like an additional task.	YW was very motivated; this motivation was contagious and persuaded adolescents to participate in the intervention.	YW was very motivated.	Mixed motivation of adolescents to participate in the intervention.
Other	Smoking is less present in YSW organisation. YW felt like the only one in the team invested in the project. Strong bond of trust between YW and adolescents.	Some interviewed adolescents have parents or friends who smoke, one of them already tried smoking. Strong bond of trust between YW and adolescents.	YW does not have a team to rely on, making implementation harder.	Vaping is a more urgent problem according to YW.	Low urgency to work on smoking. According to adolescents, they have bigger problems than smoking. YW was sometimes alone during implementation, making it harder to stay with the group when they do the activities.	Smoking is an urgent problem in this organisation due to the high prevalence. Strong bond of trust between YW and adolescents.	Healthy environment, which makes smoking not very present or urgent to work on. Very structured activities: one hour of football per week.	No organised activities, except during holidays. Adolescents can do whatever sport they like. Almost no smokers among adolescents, making smoking less urgent. Organisation does not have its own location. Adolescents trust the YW. YW did not feel supported by her colleagues.
Implementation								
Dose delivered	All components were implemented, except for the tips & tricks.	Only two components were implemented: one game and mood boards.	Only two components were implemented: mood boards and one game. YW did not see the relevance of other IV components.	Only mood boards were implemented.	Four components were implemented: mood boards, two games and the challenge.	All components were implemented.	Only two components were implemented: elements from the two games were used to create a new game including football.	Two components were implemented: mood boards and a game.
Fidelity	YW made many adaptations to make the intervention more feasible. Low fidelity because of many adaptations, but all key elements remained. YW added an activity and involved youth in a participatory way.	High fidelity for implemented components. Mood boards were used as conversation starter.	Fidelity was low.	YW used mood boards to create an educational moment and talk about smoking with adolescents.	Fidelity was relatively high for implemented components, YW made some small adaptations.	High fidelity.	Fidelity was very low.	High fidelity for implemented components.
Dose received among adolescents participating in process evaluation study	Only some interviewed adolescents received IV.	Interviewed adolescents did not receive IV; only one had a conversation about smoking during implementation period.	Interviewed adolescents did not receive IV.	Adolescents were not interviewed in this organisation.	Most interviewed adolescents did not receive IV.	None of the interviewed adolescents received IV, but some of them had a conversation about smoking with a YW.	Only some interviewed adolescents received the IV.	Only some interviewed adolescents received IV.
Feasibility and ease of implementation	Intervention was perceived as less feasible in its current state, because of too much focus on education. Implementation was not easy. YW needed extra explanation from researchers during implementation.	Intervention was perceived as feasible. Intervention was perceived as easy to implement; additional support was not needed.	Implementation was difficult. YW found it hard to intervene when adolescents were in a group.	Intervention was perceived as less feasible, because of too much focus on education. Implementation did not go well; YW was not able to motivate adolescents to participate. YW discontinued implementation because of resistance from adolescents.	Intervention was perceived as feasible. Implementation was not hard, nor very easy. Implementation sometimes felt as an additional task.	Intervention was perceived as feasible. Implementation was easy.	Intervention was less feasible for this organisation because of the sports context. Implementation was hard; many adaptations were needed to make the intervention fit in the football lessons.	Intervention was less feasible for this organisation. Implementation was hard; sport context made it harder to implement. Hard to combine regular work with implementing the intervention.

*RA* recreational activities, *SA* sport activities, *YW* youth worker

### Perceptions concerning KickAsh! and its impact

In general, the intervention was well accepted by both youth workers and adolescents. They positively described the intervention components, calling them appealing, fun, and interesting. The use of games and visually engaging materials, such as colourful mood boards, enhanced acceptability among most adolescents. Some youth workers and adolescents provided feedback on how the intervention or certain components could be improved or adapted. Others felt the intervention was already well-developed and required few changes.

*“I liked it (the KicksomeAsh!-challenge) too*,* on the one hand because I’m creative*,* but also because it allows you to learn about other people’s opinions about smoking.” – Adolescent*,* RA*.

*“And how it (the mood boards) looks*,* I would rather read it than a boring book. It seems interesting to me.” – Adolescent*,* RA*.

Several adolescents indicated that, if given the freedom to design a new smoking-related project, they would adopt more creative approaches, such as theatre or film. They also suggested using social media campaigns or creating collection points where youth can exchange cigarettes for a reward. Youth workers suggested improvements to the intervention, including organising a theme day or week instead of a smoke-free camp, as this could potentially leave a stronger impact on adolescents. Others recommended inviting an external speaker or expert (by experience), believing this would leave a stronger impression on adolescents than delivering the health message themselves.

*“I think I would make a movie or something*,* but not with too many facts*,* more fiction and raising awareness.” – Adolescent*,* RA*.

Despite the intervention being well-accepted, many youth workers indicated that they neither observed nor expected an immediate effect on adolescents’ smoking behaviour. Some doubted that the intervention would ever lead to behavioural change, arguing that smoking can never be fully addressed due to the multitude of influencing factors. Nevertheless, many youth workers did perceive an impact on raising awareness (both in adolescents and youth workers) and on making smoking a discussable topic. According to them, the intervention was particularly useful in opening up conversations about smoking by lowering the threshold and facilitating more open dialogue. Especially the mood boards were frequently mentioned and used as conversation starters, even in organisations where implementation was challenging.

*“Yes*,* that conversation was mainly initiated by the toolkit. So you are succeeding in your aim there. […] In making it discussable*,* that’s also what I said at the beginning*,* it’s not a sexy topic.” – Youth worker*,* RA*.

*“Those mood boards just hang there*,* they (adolescents) look at them*,* and you can already start the conversation.” – Youth worker*,* RA*.

Some youth workers raised concerns about the intervention’s sustainability. For instance, they noted that repeatedly using the same games is challenging over time. To enhance sustainability, they suggested emphasising the ‘tips & tricks’ component of the intervention, as it could embed smoking prevention within the organisation. Youth workers also emphasised the importance of sustained engagement with the topic over a longer period to achieve a meaningful impact. One of the youth workers (RA) explained: *“Bringing it up a few times will have an effect. It will contribute. But if you really want to say ‘this must ensure smoking prevention’*,* then it is something that needs to be much more integrated into your organisation.”*

### Preferences and adaptability

In several YSW organisations, adolescents discussed whether they preferred playing a game or having a conversation about smoking. These discussions appeared relevant, as one of the main perceived effects of the intervention was that it opened up conversations about smoking. If adolescents themselves favour dialogue, the necessity of developing numerous games becomes questionable. Our findings suggest that preferences varied: some adolescents believed a conversation was better, while others felt that games were more accessible or engaging. Adolescents noted that age may influence these preferences, with older youth favouring conversations and younger ones being more receptive to games. Some youth workers similarly believed that adolescents prefer conversations over games, considering them easier to facilitate and more impactful. Adolescents in these organisations often agreed, indicating a shared preference for conversation-based approaches. Notably, these views were frequently expressed by youth workers who faced significant challenges during implementation. A contradictory observation was that one such youth worker, while advocating for conversations, also stressed the importance of engaging adolescents with the topic unconsciously or indirectly, given their ‘reluctance’ to discuss smoking.

*“In general*,* I like*,* um*,* discussing things. I enjoy doing that*,* talking with everyone in a group.” – Adolescent*,* RA*.

*“If there are children who want to talk about it but don’t have anyone at home to talk to*,* they can always talk to someone here (at the YSW organisation). […] I think both (conversation and games) are good because in a game they can learn about what’s not good and smoking in a more fun way than just talking about it.” – Adolescent*,* RA*.

Some youth workers perceived the intervention as too schoolish or too education-focused. One youth worker explained that this made the intervention less feasible for their organisation, as they deliberately avoid adopting a school-like approach because many of their adolescents experience difficulties in school. However, this perception warrants nuance, as these organisations often implemented only one or two components of the intervention, typically those aimed at increasing knowledge (e.g. the mood boards or the tobacco plantation game), and fidelity was generally low. In one YSW organisation, even a youth worker involved in the co-creation process felt the intervention was too education-focused. Nevertheless, he adapted the intervention to better align with his “style” and the organisation’s working methods, creating a new game by using various elements from the original intervention and adding an activity which, in his view, placed greater emphasis on experiential learning. Although fidelity was low, he retained all key components necessary to address the intended determinants.

*“I would not recommend it (the intervention) because*,* for me*,* the emphasis during the games is very much on education. And*,* um*,* that doesn’t suit me and my coaching style*,* it doesn’t suit our organisation.” – Youth worker*,* RA*.

One youth worker noted that implementation will vary between youth workers according to their personal working style and the organisational context. She viewed the intervention as a good basis, highlighting its flexibility and adaptability to suit different organisational needs, interests of youth workers or youth, and levels of engagement with the topic. Other youth workers confirmed that the intervention was easy to implement and aligned well with their YSW organisation, particularly due to its use of games and creative activities. However, some found it difficult to adapt the intervention and reported that making the intervention feasible for their setting required considerable effort. These youth workers generally experienced the implementation process as challenging, at least for certain components of the intervention.

### Smoking status of youth workers

The smoking status of the youth workers appeared to influence the implementation of the intervention. In organisations where youth workers were current smokers, implementation tended to be less successful (i.e. one of these organisations even withdrew) and fewer intervention components were delivered. Even when youth workers were highly motivated concerning the intervention, they expressed discomfort with promoting not to smoke while being smokers themselves. This led to the use of alternative approaches, such as sharing personal experiences and having conversations with youth about smoking rather than delivering the intervention as intended. One youth worker considered his smoking unproblematic if not done in adolescents’ presence, whereas others feared it undermined the credibility of the non-smoking message and the messenger. Adolescents emphasised the importance of youth workers setting a good example, and appreciated it if they did not smoke in or around the YSW organisation.

*“I thought it was a bit hypocritical of myself that I was proclaiming not to smoke […] I found that a bit difficult and that’s actually why I think I may have unconsciously not used that much of your intervention.” – Youth worker*,* RA*.

*“I think it is good that there is no smoking in or around this building (the YSW organisation)*,* because it has to be very clear that it is not good for you. What they (youth workers) do outside of [YSW organisation] is their own decision. […] But they can not do it in front of small children*,* they are an example.” – Adolescent*,* RA*.

Youth workers who had previously smoked but were non-smokers during the intervention period perceived their experience as a facilitator for implementation, enabling them to engage authentically with the intervention without conflicting messages. One youth worker (RA) explained: *“That (having smoked in the past) might give me some experiences to share with the kids*,* like how many times I’ve tried to quit.”* Conversely, youth workers who had never smoked reported difficulties in implementation, feeling unable to relate to the experience of smoking and uncertain about how to discuss it meaningfully with adolescents. One youth worker even questioned the relevance of the intervention for himself due to this lack of personal experience. These youth workers suggested that additional information on adolescent smoking, such as facts and context, could enhance their ability to implement the intervention.

*“Yeah*,* well*,* I don’t smoke myself*,* […] I’ve never smoked in my life*,* so I think that it was*,* well*,* less relevant for me to speak to young people about it.” – Youth worker*,* RA*.

### Skills and motivation of youth workers

Youth workers’ experience and skills, such as group management, exercising a certain level of authority, and the ability to engage young people in unfamiliar activities or deliver them in a fun way, appeared to influence the implementation process. As one youth worker (RA) stated: *“At first*,* it’s always the case that if they (adolescents) don’t know something*,* they don’t want to do it*,* so they need to be given a bit of time. And if you have someone who communicates about it in a motivated way*,* or who is enthusiastic about it*,* then it becomes easier.”* This quote also highlights the significance of motivation and the youth worker’s attitude in implementing the intervention. Youth workers who were genuinely motivated or felt committed to the project were more likely to adapt the intervention to their context, perceived it more positively, and welcomed it within their organisation. Conversely, those who doubted its relevance or potential impact were less likely to implement the intervention adequately. This motivation appeared to be influenced by multiple factors, including the smoking status of youth workers, as discussed in the previous section. Still, even if feasibility was questioned, many of the youth workers mentioned that the intervention supported them in addressing smoking by providing them with new strategies, increasing their knowledge, and offering insight into the situation of their YSW organisation. Some youth workers even reported capacity building, including improved skills to tackle smoking more sufficiently.

*“The fact that there are games around it (smoking) is useful because it gives you something to hold on to*,* and it also makes you think: ‘aren’t there more creative ways to deliver this message than just*,* um*,* raising awareness through education and giving those kids a PowerPoint presentation about how bad smoking is?’ It stimulates your creativity*,* so it lowers the threshold and encourages you to get creative with it.” – Youth worker RA*.

Nevertheless, several youth workers indicated a need for additional support to enhance their skills in implementing the intervention. Notably, these were mainly youth workers who perceived the implementation process as difficult. They highlighted the importance of a contact person or someone who could visit their organisation to provide training concerning the intervention. Other youth workers also suggested creating a FAQ page on the website or instructional videos to offer guidance and address common questions related to implementation.

*“I think it would be much more accessible if the youth workers who are supposed to supervise the game had seen it being played by you (the researchers) at least once*,* or had actively experienced the game themselves*,* or you could have made a video of it.” – Youth worker*,* SA*.

### Organisation and structure of YSW organisations

The aim of the YSW organisation and the types of activities they offer influenced the implementation of the intervention. In organisations with a focus on sports, youth workers found it more challenging to implement the intervention compared to organisations offering leisure time or other activities, as the intervention components were less feasible in this context. For example, it was difficult to integrate some of the activities into structured football lessons, or they lacked the appropriate space or materials to implement them. As a result, youth workers had to adapt the intervention components to fit their setting, which in turn affected implementation fidelity. Adolescents also expressed concerns, noting that they attend the organisation primarily to engage in sports and feared that time spent learning about smoking would reduce opportunities to play football. One of the youth workers suggested adding examples on how the games or other activities could be translated to different contexts, e.g. if the organisation is more sport-focused.

*“Some of the components actually were not realistic for our setting. […] I had to reinterpret some things to make them applicable within our setting*,* […] because those children do come to us for sports.” – Youth worker*,* SA*.

*“We come here to play football*,* not to learn (about smoking).” – Adolescent*,* SA*.

Logistical and structural barriers were evident in some YSW organisations. For instance, when organisations lacked their own physical space, it was more difficult to implement certain components of the intervention, such as the mood boards or the KickSomeAsh-challenge. Time constraints also posed a barrier, with some youth workers reporting a lack of sufficient time alongside regular work or underestimating the time required to prepare the activities. Additionally, limited team support hindered implementation for some. In cases where youth workers had no team to assist with activities, or where a team existed but did not share their commitment to tackle smoking, they struggled with having to manage the intervention alone. Team support emerged as a crucial factor, with one youth worker expressing that he felt like the only one invested in the project, despite believing that the topic should be embraced collectively by the entire team.

*“You have your regular work*,* and it’s an extra activity […] in addition to all the other projects you already have […] and then it also depends on the number of people or the number of employees or interns you have at that moment whether it*,* yeah let’s say*,* fits or whether there is room for it.” – Youth worker*,* RA*.

Lastly, YSW organisations are environments where young people are granted considerable freedom and autonomy. Unlike, for example, school settings, it is more difficult to direct or compel adolescents to participate in activities. Some youth workers identified this unconditional and voluntary nature of YSW as a barrier to implementation, as it limited their ability to engage adolescents in the intervention. A youth worker (RA) explained that *“People come and go when they want here. And to really build something up like that (the intervention) or to create a permanent group to get started with it […] is really not that easy for us.”*

### Addressing the topic of ‘smoking’ at YSW organisations

When adolescents were asked whether they would like to ‘learn’ about smoking within the YSW organisation, many responded negatively, explaining that they attend the organisation to have fun, play, or engage in sports. However, when reframing the question and asking whether they would be interested in talking about or playing a game related to smoking, adolescents were generally more enthusiastic and open to participating. Some adolescents even preferred addressing the topic in YSW organisations rather than at school, which they perceived as more boring. Others noted that the bond with youth workers differs from that with teachers, which could enhance the implementation and impact of the intervention. Still, adolescents stressed not to overload them with the topic of smoking. While willing to engage with it occasionally, they did not want it to dominate their experience at the organisation. Others preferred to keep all smoking-related discussions and activities within the school context.

*“I wouldn’t mind it once or twice. But if every time I come here*,* we have to talk about smoking and so on*,* I would get a bit fed up with it. I don’t think I would come here as often then*,* because I come here after school to play and chill.” – Adolescent*,* RA*.

*“I think school is the best place to talk about these things (smoking) because you’re already there to follow lessons in a serious context. If you are already serious about something*,* then you also have a more serious attitude*,* that is the best time to talk about it.” – Adolescent*,* SA*.

In some cases, youth workers struggled to motivate adolescents to participate or remain engaged in the intervention. This feeling of resistance emerged as a key barrier to continued implementation. Youth workers often attributed such resistance to the topic itself, describing smoking as unattractive or even taboo among adolescents. One youth worker explained that insisting too much on smoking could jeopardise their bond of trust with adolescents. Interestingly, this bond of trust, identified by most participants as a facilitator for implementation that fosters a safe space in which adolescents feel heard and are more willing to share personal experiences, also functioned as a barrier. Some youth workers were hesitant to implement the intervention out of concern that it might compromise their relationship with the adolescents.

*“You can keep investing in it (the intervention) to keep doing it*,* but at a certain point we… look*,* we didn’t think it was worth it […] that it was not relevant to really continue with that.” – Youth worker*,* RA*.

During the focus groups, adolescents did not explicitly describe smoking as taboo. One participant mentioned that she found it a strange subject to talk about, while others expressed that they already knew enough about smoking and were uninterested in learning more. Some adolescents laughed frequently or appeared hesitant to speak openly during the interviews, suggesting a degree of discomfort with the topic. This subtle discomfort could indicate that, although not perceived as taboo, smoking remains a sensitive or awkward subject for some adolescents. However, this behaviour could also reflect a lack of relevance: for adolescents who have no intention to smoke or who hold strongly negative attitudes towards smoking, the topic may feel boring rather than uncomfortable.

*“It would be embarrassing. Because imagine if they ask you: “Have you ever smoked?” What are you going to answer? […] It’s a strange topic to talk about. […] And you also would have to talk about it with people you don’t know very well. […] Personally*,* I wouldn’t talk about it or anything like that*,* or to anyone.” – Adolescent*,* RA*.

Another barrier to implementing the intervention was the urgency to address smoking initiation in the YSW organisation. In organisations where smoking prevalence among youth was low or absent, youth workers often deprioritised the intervention, focusing on more pressing concerns. One of the adolescents also indicated that smoking is not the most important problem they are facing. Notably, vaping was mentioned in almost all organisations as a current and maybe even more urgent problem than cigarette smoking. Nonetheless, youth workers acknowledged the value of smoking prevention and recognised that early intervention could be beneficial. Clarifying that the intervention aims to prevent smoking initiation may help increase its perceived relevance.

### Participation and the co-creation process

Whether a YSW organisation participated in the co-creation process influenced the implementation process. Youth workers involved in developing the KickAsh!-intervention were more invested in the project and made greater efforts to implement it, despite encountering barriers, such as a sport-focused context or limited manpower. Attendance at the co-creation camp was particularly beneficial, as these youth workers were already familiar with the activities and understood how to adapt them to their context, making implementation easier and less cognitively demanding. One youth worker noted that her prior experience reduced challenges and even enabled her to support colleagues. Another youth worker reported an increased motivation because of his involvement, as well as a strong sense of ownership. He believed that the co-creation project had a greater impact than, for example, playing a few games during training, as it spanned a longer period and provided a meaningful and positive experience for himself and adolescents.

*“The advantage was that we experienced the camp. […] So I already had an idea of what would work in our setting and what wouldn’t. To be honest*,* I didn’t have to think too much about it […] it was already clear to me*,* because we also had that co-creation process*,* we were involved in it.” – Youth worker*,* SA*.

Some youth workers suggested implementing parts of the intervention in a more participatory way. Interestingly, these suggestions mainly came from youth workers who were involved in the co-creation process. For example, one youth worker involved adolescents in the intervention’s policy component, emphasising the importance of youth support to ensure policy effectiveness. Other youth workers proposed involving adolescents in creating a new game about smoking. Therefore, the intervention could lead to co-creation within the organisation, potentially increasing its impact by strengthening group support for non-smoking. Such participatory approaches may also enhance feelings of belonging and reinforce a collective smoke-free identity among adolescents.

*“I think you could involve some of the older kids*,* for example*,* to develop a game together. […] And I think that could have been good for the sense of togetherness and to be more aware of the importance of being smoke-free as a group.” – Youth worker*,* RA*.

## Discussion

This study evaluated the implementation process of the KickAsh!-intervention across various YSW organisations, with a particular focus on how the implementation unfolded in real-world settings. The findings indicate that numerous factors (related to the adolescents, the youth workers, the YSW organisation, and the intervention itself) influenced the implementation of the intervention and interacted in complex ways, creating a unique context within each organisation. These contextual differences shaped how the intervention was implemented and perceived by youth workers and adolescents. As a result, substantial variability in the implementation of the intervention was observed across organisations, with only one organisation implementing all components as intended. This variability constitutes a central finding of the study and illustrates the strongly context-dependent nature of delivering complex interventions in practice. Importantly, such variability may have complicated comparisons across YSW organisations and offers a plausible explanation for the absence of significant effects (except for coping planning) in the accompanying effectiveness study [29]. Furthermore, the outcomes included in the effectiveness study may not have captured all changes triggered by the intervention in practice. While many youth workers did not perceive a direct influence of the intervention on adolescents’ smoking behaviour, they consistently noted its role in raising awareness, facilitating open dialogue, and lowering the threshold to address smoking. The intervention also supported youth workers in tackling this topic within their organisation. These findings suggest that the intervention may have influenced other relevant outcomes, such as awareness or communication, which were not measured in the effectiveness study [29].

Furthermore, the process evaluation study was guided by the MRC framework, which emphasises the importance of developing a theory of change (ToC) to explain an intervention’s underlying mechanisms [11]. As part of this study, a ToC was developed before the evaluation (see Supplementary Material 2) and subsequently tested and refined based on the findings (see Supplementary Material 7). This refined ToC outlines the mechanisms supporting successful implementation in the YSW context and adolescents’ behavioural change, and can inform further refinement of the KickAsh!-intervention as well as future intervention research in YSW settings. Reflecting on the ToC also helps situate possible explanations for the lack of observed effectiveness [29]. The results of the process evaluation suggest that inconsistent implementation and limited fidelity played a central role, alongside the possibility that effects occurred on unmeasured determinants (e.g. increased awareness) and broader contextual challenges within the highly diverse YSW sector. Together, these findings point to a combination of interacting factors, conceptualised as derailers and support factors in the ToC, rather than a single cause. Implementation challenges appear particularly influential, potentially outweighing limitations in intervention content, as both youth workers and adolescents generally evaluated the content positively despite differing preferences across components. Given the diversity of the YSW context, where activities, needs, and preferences vary widely, and considering that youth workers were encouraged to adapt the intervention to their organisational context, it seems reasonable to conclude that the content itself was not necessarily the primary issue. However, it remains difficult to draw firm conclusions. Because the intervention was not implemented consistently or in full, we cannot determine with certainty whether the content would have been effective under optimal implementation conditions.

Next, an important conclusion from this study is that youth workers are key to the implementation of the intervention. Their personal characteristics, working style, skills, attitudes, and the value they attach to smoking prevention all influence the implementation process. For example, a highly motivated youth worker who smoked felt less credible when addressing smoking prevention, which hindered implementation. This highlights the central role of the implementer, a finding consistent with existing research on implementation processes. Several implementation frameworks, such as the Consolidated Framework for Implementation Research (CFIR) [31] and the Promoting Action on Research Implementation in Health Services (PARiHS) framework [40, 41], emphasise the importance of facilitation and the individuals involved in the implementation process. These frameworks identify specific characteristics of implementers, such as knowledge and beliefs about the intervention, self-efficacy, stage of change, or facilitation style, as critical factors influencing implementation success. This suggests a need for greater attention to the development of implementation skills among implementers, with a focus on strengthening and expanding these capacities to ensure the effective delivery of health-promoting interventions. In the KickAsh!-intervention, this was addressed by providing a manual and recommendations regarding the sequence of intervention components. Moreover, before implementation, a preparatory meeting was organised with youth workers to explain the intervention and clarify expectations. Nevertheless, some youth workers struggled with implementation and expressed a need for more support during the process. They suggested more concrete examples concerning implementation in their specific context, or a contact person or someone who could visit their organisation for guidance. However, while personalised support could improve implementation, it also poses practical challenges, is difficult to sustain over time and limits the potential for broad dissemination.

Another way to reduce implementation barriers is to involve potential implementers from the start. Research underscores the importance of engaging implementers in developing implementation strategies [22]. For KickAsh!, youth workers were involved in the co-creation process, aiming to incorporate their perspectives and suggestions into the intervention and implementation design. Still, many youth workers who ultimately implemented the intervention encountered substantial challenges, raising the question of whether the level of involvement was sufficiently intensive. Indeed, most co-creation sessions focused on developing intervention components, with only two addressing implementation-related aspects [26]. This limited emphasis may have reduced the depth of engagement and youth workers’ contribution to the implementation. Future intervention development efforts may benefit from more extensive and structured involvement of potential implementers. Additionally, limited attention was given to using a structured framework for implementation planning. Although the Intervention Mapping (IM) protocol [22] was followed during development of the KickAsh!-intervention, considerably less time and emphasis were placed on Step 5, which focuses on planning for adoption, implementation, and maintenance. This underinvestment may have contributed to the implementation challenges experienced by youth workers.

As mentioned earlier, only one organisation implemented all intervention components as intended, raising important questions regarding the overall feasibility of the intervention. In some settings, the intervention was perceived as highly feasible, while others required adaptation to align with their specific context. For example, implementation within sport-focused contexts proved more challenging. These organisations often lacked the necessary resources or time to carry out the activities as intended, such as having only one hour for a football lesson. This is particularly noteworthy given that one of the YSW organisations that participated in the co-creation process was a sport-based organisation. It could be expected that this involvement would have led to a more tailored intervention for sport settings. However, it is somewhat understandable that KickAsh! was not fully adapted to the sports context, as several intervention components were co-created in preparation for the co-creation camp [26]. These components were added to the intervention based on youth feedback and enthusiasm, but were not specifically designed with sport-focused organisations in mind. Interestingly, the youth worker from the involved sport-based organisation still perceived his participation as valuable, as it helped him anticipate what might be feasible within his organisation. Furthermore, it should be acknowledged that the sports context may in itself function as a more preventive environment for substance-use behaviour, such as smoking, as sport is often associated with a healthy lifestyle, raising the question of whether it is needed to implement interventions in this type of YSW. However, sport-focused YSW organisations, often referred to as sport-for-development, have a role that extends beyond merely offering sport activities [21, 42]. They explicitly aim to provide additional support to adolescents across multiple life domains, including health, socialisation, and social inclusion [21, 42]. In doing so, these organisations strongly reflect the social and emancipatory principles underpinning YSW. Consequently, concrete interventions that support youth workers operating in these settings remain highly relevant, as they can help youth workers to more fully enact this broader supportive role.

Still, variation in feasibility of the KickAsh!-intervention across organisations is not entirely unexpected. Co-created interventions are typically localised and tailored to the specific context in which they are developed [25, 43]. Our findings confirm that YSW organisations differ in aims, methods, and needs, making it unrealistic to expect an intervention co-created with two organisations to fit all others. On the other hand, co-creating an intervention from scratch for every individual organisation is impossible, given the time- and resource-intensive nature of co-creation [25, 44]. One potential solution could therefore be to embed adaptability within the current intervention by providing youth workers with guidelines for context-specific adjustments, ideally in a participatory way with adolescents. This could be particularly effective in YSW organisations, where participatory methods are widely embraced [17], and youth workers often have experience in designing or modifying games and activities. Our findings also support the need for adaptability. For example, some of the youth workers used the mood boards and statement cards as conversation starters. Consequently, if a youth worker observes that a conversation-based approach resonates more with their group than a game-based activity, they could adjust the intervention accordingly, provided that the key components of the intervention (i.e. those elements that target determinants) remain present. This flexibility allows youth workers to tailor the intervention to their specific needs, preferences, and what works best in their unique context. Moreover, empowering youth workers to adapt the intervention themselves may enhance feasibility and even ownership, while maintaining the integrity of the original intervention.

To translate these insights into actionable directions, we summarise recommendations for future research and intervention development in Table 3.

**Table 3:** Recommendations for future interventions in youth social work based on the process evaluation of KickAsh!

Recommendations for future research and intervention development in YSW:
• Develop a context‑sensitive implementation strategy in close collaboration with youth workers through co‑creation.
• Embed adaptability within the intervention design, allowing it to be tailored to diverse YSW contexts.
• Include concrete good practices and examples in the intervention guidelines to illustrate how components can be adapted while preserving their core elements.
• Prioritise participation and emancipation of adolescents, ensuring that interventions align with core social‑work values.
• Provide additional support for youth workers on how to address smoking, particularly when it is perceived as a sensitive or unattractive topic.
• Integrate smoking prevention into the broader approach and organisational policy of YSW settings.
• Utilise creative, engaging, and low‑threshold methods to address smoking in an accessible way.
• Develop a context‑specific Theory of Change to support implementation; the model provided in Supplementary Material 5 can serve as a starting point or illustrative example.

This study entailed several limitations. First, the unconditional nature of YSW organisations made it difficult to recruit adolescents who had actually received the intervention. Many of the adolescents interviewed during the focus groups indicated only partial or no exposure to the intervention. This is closely linked to the flexible attendance structure of YSW organisations, where adolescents can come and go freely, reducing the likelihood that they were present during the delivery of the intervention. To proactively address this, process evaluation questions were added to the effectiveness questionnaire to complement the qualitative data. However, these data were similarly limited, as the same challenge was encountered: few adolescents who had received the intervention completed the questionnaire. During focus groups, when adolescents indicated they had not received certain components, we explained the content and asked whether they would be interested in participating in such activities. This allowed these adolescents to contribute to the focus group, even though they had not been exposed to the intervention. However, this approach proved challenging, as it can be abstract for youth to imagine an activity they have not experienced firsthand. Additionally, adolescents who received the intervention may not have been present on the day the focus group was organised, further limiting the ability to gather comprehensive data. To improve future data collection, it may be beneficial for implementers to keep a record of participants who engage with the intervention, allowing researchers to contact them in advance to organise focus group interviews. Another option could be to introduce short evaluation forms for adolescents to complete immediately after each intervention component. A second limitation is that interviews and focus groups were conducted only after the implementation period. Given the number of barriers identified, collecting data during implementation may have yielded richer insights, particularly as the four-month period may have challenged participants to recall their experiences with specific intervention components. Although follow-up conversations with youth workers were organised during implementation, many indicated that they had not yet implemented most components, which limited meaningful reflection at that stage. Furthermore, the logbook provided as an implementation diary was used consistently by only a few youth workers. Future research should therefore consider more accessible and engaging methods to support youth workers in documenting implementation. Next, adolescents were sometimes distracted, disengaged, or playful during the focus groups, which occasionally hindered the flow of the conversation. This may relate to the leisure-oriented nature of YSW, where adolescents mainly come for enjoyment, making participation in group discussions less appealing. Although we used interactive methods to increase engagement, some still struggled to stay focused, which may have affected data quality. Another limitation concerns the potential for social desirability bias during the interviews and focus groups. Youth workers may have provided less truthful responses to avoid disappointing the researchers, while adolescents’ answers may have been influenced by peer presence. Still, several measures were taken to mitigate this risk. Confidentiality was emphasised at the outset, and participants were reassured that there were no right or wrong answers and that any opinion was relevant. With adolescents, alternative, more interactive interview methods were used (e.g. stickers to express opinions) to facilitate more honest and open responses. For youth workers, prior informal visits to their organisations may have strengthened trust, thereby encouraging more honest responses. A final limitation concerns the dropout of three YSW organisations before implementation, which may have introduced selective attrition leading to potential selection bias. As no interviews were conducted within these organisations, insight into organisation-specific barriers to implementation is lacking. Reported reasons for withdrawal were a lack of time and personnel, as well as limited motivation. Another organisation withdrew during implementation, but an interview with the youth worker was conducted and provided insights into discontinuation. However, a focus group with adolescents was not feasible, leaving their perspectives on disengagement unrepresented.

## Conclusion

This study demonstrates that successfully implementing a co-created smoking prevention intervention in the YSW context requires consideration of the interaction between individual, organisational, and contextual factors. Youth workers, as primary implementers, play a pivotal role in this process. Strengthening youth workers’ skills and capacity to deliver the intervention, engaging and involving them thoroughly in the development of implementation strategies, and embedding opportunities for flexible, context-specific adaptation of the intervention can enhance the feasibility, impact, and sustainability of similar interventions in YSW organisations.

## Supplementary Information

Supplementary Material 1.

Supplementary Material 2.

Supplementary Material 3.

Supplementary Material 4.

Supplementary Material 5.

Supplementary Material 6.

Supplementary Material 7.

## Data Availability

The datasets used and/or analysed during the current study are available from the corresponding author on reasonable request.

## Abbreviations

CFIR: Consolidated Framework for Implementation Research
COREQ: Consolidated criteria for Reporting Qualitative research
IM: Intervention Mapping
MRC: Medical Research Council
PARiHS: Promoting Action on Research Implementation in Health Services
QCA: Qualitative Content Analysis
RA: Recreational Activities
SA: Sport-based Activities
ToC: Theory of Change
YSW: YSW
