# Supplementary material for: Preventing smoking initiation among vulnerable adolescents: a process evaluation of the KickAsh!-intervention in Flemish youth social work settings
Source: Arch Public Health. 2026 Jun 5;84:180. doi: 10.1186/s13690-026-01972-6 (PMC13455221; doi:10.1186/s13690-026-01972-6)
Supplement: Supplementary file 1 [file 13690_2026_1972_MOESM1_ESM.pdf]

Appendix A

Overview of the KickAsh! intervention

Intervention component	Content	Core components	Targeted determinant (D) / environmental factor (EF)
Smoking policy	Checklist to delineate the current smoking policy, supplemented by tips and tricks on how to develop or improve this policy. Organisations receive a 'smoke-free' label when meeting all requirements.	– Checklist	– Organisation (EF)<br>– Policy (EF)
Tips & tricks for youth workers	Tips and tricks concerning how to be a role model, inoculation theory, assertiveness and other life skills, planning coping responses and self-efficacy of adolescents.	– Tips & tricks	– Social influence (EF)<br>– Self-efficacy (D)<br>– Skills (D)
Smoke-free games	Instructions and required materials to play two games: 'tobacco plantation game' and 'operation smoke-free'.	– Statement cards<br>– Cases<br>– ID-cards	– Knowledge (D)<br>– Attitude (D)<br>– Risk perception (D)<br>– Subjective norm (EF)
Design a smoke-free game	Guidelines how to make an evidence-based smoke-free game customised to the organisation, with the possibility to upload this to the website.	/	/
Mood boards	Visual mood boards which can be placed on different spots in the organisation, addressing three themes: smoking and health, smoking and the climate, smoking and child labour.	– Mood boards	– Attitude (D)<br>– Risk perception (D)<br>– Knowledge (D)
Smoke-free camp	Script and necessary materials for going on a 2-3 day smoking prevention-themed camp.	Combination of the other intervention components	– Knowledge (D)<br>– Risk perception (D)<br>– Attitude (D)<br>– Social influence (EF)<br>– Subjective norm (EF)
Kick some Ash!-challenge	Organisations can attend the challenge by creating their own smoke-free logo, the best logo wins a price.	– Creating smoke-free logos	– Risk perception (D)<br>– Attitude (D)<br>– Self-efficacy (D)<br>– Skills (D)<br>– Organisation (EF)<br>– Subjective norm (EF)
