# Supplementary material for: Preventing smoking initiation among vulnerable adolescents: a process evaluation of the KickAsh!-intervention in Flemish youth social work settings
Source: Arch Public Health. 2026 Jun 5;84:180. doi: 10.1186/s13690-026-01972-6 (PMC13455221; doi:10.1186/s13690-026-01972-6)
Supplement: Supplementary file 2 [file 13690_2026_1972_MOESM2_ESM.pdf]

Interview Guide Adolescents (ENG version[1])

At the beginning of the session, the participants are asked whether they prefer a regular interview/group discussion or a more active approach.

What?	Content / method	Time
Icebreaker	Briefly explain what we will do today:<br><br>"We are Kenji and Babette, two researchers from Ghent University. Between October and now, [organisation] has participated in a project on the topic of smoking. Today we will ask a few questions about this project, but we will do it in a fun way! It is important to know that there are no right or wrong answers, and you can answer honestly. If any questions are unclear, feel free to interrupt us at any time to ask about it. Participation is voluntary, meaning you do not have to answer any question you prefer not to answer. If needed, we can take a break or stop the interview at any time."<br><br>"Before we begin, we will first warm up a bit."<br><br>Depending on the group, a different icebreaker can be used:<br><br>• Briefly go around with names + what is your favorite food?<br>• Word cloud via Wooclap or Mentimeter: "Complete: smoking cigarettes is…"<br>• Spinning game: https://ambrassade.be/nl/kennis/artikel/draaien<br>• Tag game with "aaaaa": https://ambrassade.be/nl/kennis/artikel/aaaaaaaaaaaaaaaaaaaaa<br>• Ninja	10 min
Which components do you recognize?	For each intervention component, an image is provided:<br><br>• mood boards	25 min

[1] Translated with Microsoft CoPilot, original version in Dutch

What did you like/dislike?<br><br>(dose received, experience, feasibility, mechanisms of impact)	• tobacco plantation game<br>• mission smoke-free<br>• another game about smoking<br>• camp about smoking<br>• new rules about smoking (policy)<br>• conversation with a youth worker about smoking<br>• Kick some Ash challenge<br><br>Stickers are provided with six types of emojis:<br><br>• Smiling = I liked it / it was good<br>• Heart eyes = I found it nice / beautiful<br>• Nauseated = I found it ugly<br>• Thumbs up/down = I liked / didn't like it<br>• Glasses emoji = I found it interesting<br>• "I don't know" emoji = I don't recognize the component<br><br>Participants can place stickers on the different components to express their opinion. They may combine stickers.<br><br>Afterward, all components are reviewed together, and follow-up questions are asked about why they chose certain emojis and what could be improved. Questions are also asked about how the component was implemented.<br><br>For the "other game," ask which game they played.	
General opinion (extra)<br><br>(experience, feasibility, mechanisms of impact)	Each participant receives a clothespin. To indicate how they felt about the intervention overall, they attach the clothespin somewhere on their body. The higher they place it, the more positive their evaluation (e.g., if it were a score out of 10, the top of the head is 10 and the toes are 0).<br><br>→ A photo can be taken of this<br><br>Possible follow-up:	5 min

	• In what way did the intervention help (or not)?	
Statements<br><br>(context, experience, feasibility)	A line is drawn: one side represents "true/correct," the other side "false/incorrect." This can also be done by raising hands.<br><br>Statements are presented to the participants:<br><br>• I think it is good if youth workers do not smoke at [organization].<br>○ Have you ever seen a youth worker smoke?<br>○ Did they smoke between October and now?<br>○ Where/when did they smoke?<br>○ What do you think about playing a game about smoking and then seeing a youth worker smoke afterward?<br><br>• I have had a conversation about smoking with one of the youth workers of [organisation]. (if not yet discussed in the previous part)<br>○ What was the conversation about?<br>○ How did you experience it?<br><br>• I have talked about the project with someone other than a youth worker (e.g., parents, other young people, at school, etc.). (extra)<br>○ What was that conversation about?<br><br>• If someone offers me a cigarette, I feel confident saying "no."<br>○ How would you do that?<br>○ Would you find it interesting to learn more about this? How would you like to learn (e.g., game, discussion, video...)?<br><br>• I like learning about the topic of "smoking" at [organization].	10 min

	○ You could also do this at school, what do you think about that?<br>○ Are there other places where you could learn about smoking?	
Final question<br><br>(feasibility)	“Imagine you receive 10 million euros to improve the project—what would you do to make it better?”	5 min
Cool down	Have a snack (e.g., a cookie)	5 min
