# Supplementary material for: Preventing smoking initiation among vulnerable adolescents: a process evaluation of the KickAsh!-intervention in Flemish youth social work settings
Source: Arch Public Health. 2026 Jun 5;84:180. doi: 10.1186/s13690-026-01972-6 (PMC13455221; doi:10.1186/s13690-026-01972-6)
Supplement: Supplementary file 3 [file 13690_2026_1972_MOESM3_ESM.pdf]

Interview Guide Youth Workers (ENG version[1])

Introduction (10 minutes)

Hello! Thank you for participating in our conversation about the KickAsh!-intervention. I will briefly explain the purpose of this interview.

For about two years now, we have been working on developing a digital platform (website) containing components that you, as youth workers, can use to organize engaging activities around the theme of "not starting smoking" with young people in your organisation. Over the past few months, you have likely carried out several activities related to smoking among youth.

With this interview, we aim to gain a better understanding of your experience with everything that has taken place in this project over the past months: what you liked and how we could improve things. We are therefore interested in how you used the components, your experience with the different components, and your opinions and thoughts about KickAsh! in general.

There are no right or wrong answers, and you can answer honestly. We will learn most from your feedback if you share your honest opinion about the collaboration with us. So if there is something you hesitate to say, that is exactly the kind of valuable information we are looking for! We have 60 minutes for this conversation, so you have plenty of time to reflect and share everything that comes to mind.

If any questions are unclear, feel free to interrupt me at any time for clarification. Participation is voluntary, meaning you do not have to answer any question you prefer not to answer. If needed, we can take a break or stop the interview at any time.

With your permission, I would like to record this conversation, as I cannot write down everything during the discussion. This way, we can analyse the conversation afterward. Certain information you provide may be used in the research, but it will not be possible to trace it back to you personally. Please be as open and honest as possible, this helps us the most.

Alright, almost ready:

- Write down participants
- Turn on the voice recorder

[1] Translated with Microsoft CoPilot, original version in Dutch

Introduction Question (context)

Before we start, we would like to ask whether you smoke. It is absolutely not a problem if you do. You do not have to answer this question if you prefer not to.

If yes:

- How often do you smoke?
- Do you ever smoke when you are at [organisation]?

INTERVENTION

General (dose delivered, implementation process, feasibility)

The KickAsh! toolkit consisted of several components (open the website to show). We will go through each component in detail shortly, but first we would like to ask a few general questions.

- To what extent were you aware that the KickAsh! project was taking place in [organisation]?
- To what extent were you aware of the content of the KickAsh! project?
- How relevant did you find the toolkit for your organisation?
  - Did you feel it suited your organisation?
- To what extent did the toolkit motivate you to address the topic of smoking within your organisation?
- Do you remember which components from the toolkit you or your colleagues/interns used in the past months?
- Overall, did you find it easy or difficult to use/implement the toolkit in [organisation]? Why easy/difficult?

Individual Components (dose delivered, fidelity, adaptations, implementation process, feasibility, experience)

Make Your Organisation Smoke-Free (Smoking Policy)

- Does your organisation have a smoking policy? **(context)**
  - What does this policy look like? What rules or agreements exist?
  - Has this policy existed for a long time, or was it developed during the past three months?
  - Is the policy properly followed? Why or why not?
- What did you think of the support in the KickAsh! toolkit for developing a smoking policy?
  - Did you use this component? Why or why not?
  - How did you implement this component?
  - What difficulties did you encounter?
  - How useful/interesting was it?
  - How could we improve this component?

Tips & Tricks for Youth Workers

- What did you think of the "tips & tricks for youth workers" section? This section provided tips on supporting young people's self-efficacy in not starting smoking (e.g., situational confrontation, assertiveness, coping plans).
    - Did you use this component? Why or why not?
        - Which tips did you use?
    - How did you implement it?
    - What difficulties did you encounter?
    - How useful/interesting was it?
    - How could we improve it?
- Over the past three months, have you had a conversation about smoking with one or more young people?
    - If yes: what was the conversation about?
    - How did you experience it?

Games – Choose a Game

- What did you think of the "choose a game" component? (games: tobacco plantation game and mission smoke-free)
    - Did you use it? Why or why not?
        - Which games did you play?
    - How did you implement it?
        - Did you use the games as-is or adapt them? How?
        - Where did you play them?
        - How often did you play them?
    - What difficulties did you encounter?
    - How useful/interesting was it?
    - How could we improve it?

Games – Create a Game

- What did you think of the support for creating your own smoke-free game?
    - Did you use this component? Why or why not?
        - What kind of game did you create?
    - How did you implement it?
        - How often did you create a game?
    - What difficulties did you encounter?
    - How useful/interesting was it?
    - How could we improve it?

Mood Boards

- What did you think of the mood boards?
    - Did you use them? Why or why not?
    - How did you implement them?

        - Where did you place them in [organisation]?
    - What difficulties did you encounter?
    - How useful/interesting were they?
    - How could we improve them?

Smoke-Free Camp

- What did you think of the support for organising a smoke-free camp?
    - Did you use this component? Why or why not?
    - How did you implement it?
        - What did the camp look like?
        - Did you use the script as-is or adapt it? How?
    - What difficulties did you encounter?
    - How useful/interesting was it?
    - How could we improve it?

Kick Some Ash! Challenge

- What did you think of the Kick Some Ash! Challenge (designing and submitting a smoke-free logo)?
    - Did you participate? Why or why not?
    - How did you approach the challenge?
    - What difficulties did you encounter?
    - How useful/interesting/fun was it?
    - How could we improve it?

Perceived Effects (adaptations, mechanisms of impact)

We are interested in whether the project had an impact on smoking behavior among youth in [organisation].

- Do you think the project helped young people not to (start to) smoke?
    - Yes:
        - In what way did it help?
        - Was there a specific component that helped most?
    - No:
        - Why do you think it did not help?
- To what extent did the toolkit help you introduce the topic of smoking to young people?
- What could we have done differently to increase the impact of the project?
- What should definitely be retained in the toolkit in the future?
- Did you notice any other changes or effects (on yourself, the young people, or other facilitators)? Which?

Closing (feasibility, sustainability)

Finally, we would like to ask a few general questions:

- What is your overall opinion of the KickAsh! toolkit (both implementation and components), and why?
- Would you recommend the toolkit to other youth social work organisations? Why or why not?
- Is there anything else you would like to add?
