# Supplementary material for: Preventing smoking initiation among vulnerable adolescents: a process evaluation of the KickAsh!-intervention in Flemish youth social work settings
Source: Arch Public Health. 2026 Jun 5;84:180. doi: 10.1186/s13690-026-01972-6 (PMC13455221; doi:10.1186/s13690-026-01972-6)
Supplement: Supplementary file 4 [file 13690_2026_1972_MOESM4_ESM.pdf]

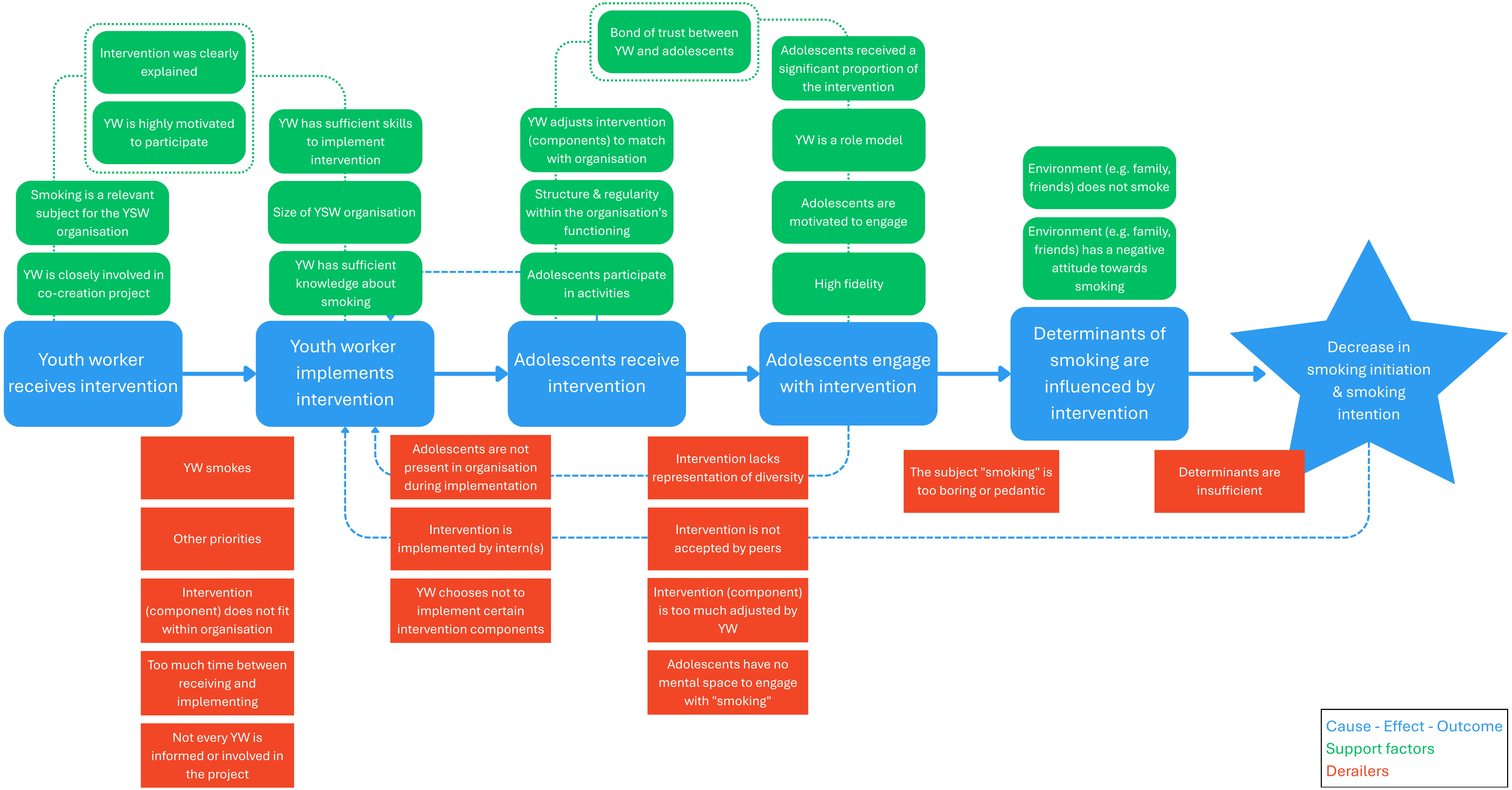
Intervention was clearly explained
YW is highly motivated to participate
Smoking is a relevant subject for the YSW organisation
YW is closely involved in co-creation project
YW has sufficient skills to implement intervention
Size of YSW organisation
YW has sufficient knowledge about smoking
Bond of trust between YW and adolescents
YW adjusts intervention (components) to match with organisation
Structure & regularity within the organisation's functioning
Adolescents participate in activities
Adolescents received a significant proportion of the intervention
YW is a role model
Adolescents are motivated to engage
High fidelity
Environment (e.g. family, friends) does not smoke
Environment (e.g. family, friends) has a negative attitude towards smoking
Youth worker receives intervention
Youth worker implements intervention
Adolescents receive intervention
Adolescents engage with intervention
Determinants of smoking are influenced by intervention
Decrease in smoking initiation & smoking intention
YW smokes
Other priorities
Intervention (component) does not fit within organisation
Too much time between receiving and implementing
Not every YW is informed or involved in the project
Adolescents are not present in organisation during implementation
Intervention is implemented by intern(s)
YW chooses not to implement certain intervention components
Intervention lacks representation of diversity
Intervention is not accepted by peers
Intervention (component) is too much adjusted by YW
Adolescents have no mental space to engage with "smoking"
The subject "smoking" is too boring or pedantic
Determinants are insufficient
Cause - Effect - Outcome
Support factors
Derailers
