# Supplementary material for: Preventing smoking initiation among vulnerable adolescents: a process evaluation of the KickAsh!-intervention in Flemish youth social work settings
Source: Arch Public Health. 2026 Jun 5;84:180. doi: 10.1186/s13690-026-01972-6 (PMC13455221; doi:10.1186/s13690-026-01972-6)
Supplement: Supplementary file 5 [file 13690_2026_1972_MOESM5_ESM.pdf]

Index process evaluation KickAsh!-intervention

Name	Description	Files	References
Context		16	203
Contextual factors	Social, cultural, economic, and political factors impacting the success or failure of the intervention in a specific context	16	92
Feasibility	the assessment of the practicality and potential successful implementation of an intervention in a particular setting; the extent to which the intervention can be successfully implemented in a given setting	11	25
Mapping	Characteristics and disctribution of a specific population	9	38
Readiness for change	the degree to which individuals or organisations are prepared to adopt and implement new interventions or changes in behaviour	11	26
Resources	Adequacy and availability of resources required for implementing the intervention	8	17
Support	the level of assistance and guidance provided by management and other stakeholders to ensure succesful implementation of the intervention; a supportive environment; intervention supports activities provided; support received within the settings	5	5

Name	Description	Files	References
Delivery	'Delivery' components measured the degree to which the co-creation process and/or intervention implementation was delivered as intended.	15	173
Barriers		6	34
Dose delivered	To what extend the intervention components were delivered	8	24
Dose received	The extend to which the target population received the intended intervention components	6	13
Exposure	The extend to which the target population is exposed to the intervention components	6	9
Facilitators		5	15
Fidelity	The extent to which an intervention was delivered as intended, according to the original program design/protocol	9	43
Adaptation	Which adaptations were made to the intervention components during delivery	8	28
Adherence	Adherence to the original plan, to guidlines, to which components were fulfilled	7	10
Other		8	35
Experience		16	283

Name	Description	Files	References
Acceptability	Extend to which the intervention was perceived as suitable, agreeable and satisfactory by the target population or stakeholders	13	61
Other		6	8
Perceptions	How various stakeholders perceived the intervention	16	212
Satisfaction	Level of satisfaction among individuals who received the intervention, who implemented the intervention	2	2
Impact	The extent to which the intervention had achieved one or more of its desired outcomes and its overall impact	16	107
Adoption	The extent to which individuals, organisations or communities decided to adopt and implement a new behaviour or practice promoted by the intervention	8	20
Capacity building	The extent to which the intervention has build capacity of individuals, organisations or communities to deliver or sustain the intervention; skill enhancement	11	37
Communication	Whether information about an intervention has been effectively conveyed to the intended audience; how well was the message understood and received, as well as identifying potential barriers or gaps in communication	4	4
Mechanisms of impact	How did the intervention produce its outcomes; how does the delivered intervention produce change	5	14

Name	Description	Files	References
Other		8	24
Policy change	The impact of changes to policies and procedures	3	4
Reach	The extent to which an intervention had successfully reached its intended target population	1	4
Maintenance	The extent to which the intervention outcomes were maintained	15	109
Future organisation	Suggestions for the future organisation of the intervention and future projects	15	96
Retention	Factors that influence the continued participation in the project	5	13
Other		9	13
Non-smoking creates safe space		2	2
Vaping		7	11
Participation		8	27
Motivation	Motivations of individuals for engaging in the project, incentives that influenced the decision to join the project and to stay engaged in the project throughout	8	20
Other		4	7
